# Screening marker genes of type 2 diabetes mellitus in mouse lacrimal gland by LASSO regression

**DOI:** 10.1038/s41598-023-34072-4

**Published:** 2023-04-26

**Authors:** Xiaoting Pei, Di Qi, Jiangman Liu, Hongli Si, Shenzhen Huang, Sen Zou, Dingli Lu, Zhijie Li

**Affiliations:** Henan Eye Institute, Henan Eye Hospital, and Henan Key Laboratory of Ophthalmology and Visual Science, Henan Provincial People’s Hospital, People’s Hospital of Zhengzhou University, People’s Hospital of Henan University, No. 7, Weiwu Road, Zhengzhou City, 450003 Henan Province China

**Keywords:** Eye diseases, Diabetes, Computational biology and bioinformatics, Biomarkers

## Abstract

Type 2 diabetes mellitus (T2DM) is characterized by insulin resistance and a relative deficiency of insulin. This study aims to screen T2DM-related maker genes in the mouse extraorbital lacrimal gland (ELG) by LASSO regression.C57BLKS/J strain with leptin *db/db* homozygous mice (T2DM, *n* = 20) and wild-type mice (WT, *n* = 20) were used to collect data. The ELGs were collected for RNA sequencing. LASSO regression was conducted to screen marker genes with the training set. Five genes were selected from 689 differentially expressed genes by LASSO regression, including *Synm*, *Elovl6*, *Glcci1*, *Tnks* and *Ptprt*. Expression of *Synm* was downregulated in ELGs of T2DM mice. *Elovl6*, *Glcci1*, *Tnks*, and *Ptprt* were upregulated in T2DM mice. Area under receiver operating curve of the LASSO model was 1.000(1.000–1.000) and 0.980(0.929–1.000) in the training set and the test set, respectively. The C-index and the robust C-index of the LASSO model were 1.000 and 0.999, respectively, in the training set, and 1.000 and 0.978, respectively, in the test set. In the lacrimal gland of db/db mice, *Synm*, *Elovl6*, *Glcci1*, *Tnks* and *Ptprt* can be used as marker genes of T2DM. Abnormal expression of marker genes is related to lacrimal gland atrophy and dry eye in mice.

## Introduction

Globally, there are 537 million people with diabetes mellitus (DM), according to the International Diabetes Federation 2021. The per capita health expenditure related to DM is $1838.4. The proportion of DM-related deaths among people under the age of 60 accounts for 32.6%^1^. Type 2 diabetes mellitus (T2DM) is characterized by insulin resistance and a relative lack of insulin secretion from pancreatic beta cells^2,3^. T2DM is the most common type of DM, accounting for more than 90% of all DM in the world^4^. Patients with poorly controlled blood glucose in T2DM can cause many complications, the severe ones being cardiovascular disease and stroke^5^. T2DM can also cause many ocular complications, the commonest being diabetic retinopathy, corneal neuropathy, glaucoma and cataracts^6^. The incidence of these conditions in T2DM patients is significantly higher than in the general population.

The extraorbital lacrimal gland (ELG) is located in the superior lacrimal fossa outside the orbit. As an exocrine gland, the ELG plays a crucial role in maintaining ocular surface homeostasis, immune protection, and corneal transparency by providing water, electrolytes, growth factors, antimicrobial peptides, and neuropeptides^7^. It is well established that DM is often accompanied by lacrimal gland dysfunction, and nearly 50% of DM patients suffer from chronic dry eye^8,9^. However, the exact timing of the onset of T2DM is also usually uncertain. As many as one-third to one-half of patients with T2DM in the population may not be identified by medical diagnosis^1^. Therefore, searching for marker genes that are associated with T2DM and lacrimal gland dysfunction will potentially provide new insights into the prediction and treatment of T2DM and ocular complications in a high-risk population.

The Least Absolute Shrinkage and Selection Operator (LASSO) regression is based on regression methods to obtain more accurate predictive algorithms. The LASSO procedure is relatively suitable for models showing high levels of multicollinearity or when certain parts of the model are automatically selected, such as variable selection/parameter elimination^10^. In addition, LASSO regression models are suitable for studies where the number of predictors is greater than the sample of test subjects. This is typically used for those analyses that are based on genetic data but not on clinically used variables^10^.

Db/db mice are leptin receptor gene-deficient mice with characteristics such as obesity, insulin resistance and hyperglycemia and are widely used as models of T2DM^11^. To find marker genes associated with lacrimal gland dysfunction accompanying T2DM, the db/db mice were used in the present study as a T2DM model. First, the differentially expressed genes (DEGs) in the mouse lacrimal gland associated with T2DM were obtained by RNA sequencing and bioinformatics analysis techniques, and then the marker genes highly associated with T2DM were further screened using LASSO regression. Multiple validation was performed to assess the efficiency of marker genes in identifying T2DM. These results will provide a theoretical basis for the prediction and treatment of T2DM and lacrimal gland dysfunction caused by T2DM in the future.

## Materials and methods

### Animals

Twenty C57BLKS/J background db/db (DB) mice aged 12 weeks were used as a T2DM model, and C57BLKS/J male mice from the same litter were used as wild-type (WT) control mice. All animals were provided by the Model Animal Research Center of Nanjing University (Nanjing, China). All animals were housed in 12 h/12 h light/dark circadian chambers with free access to water and food. All animal experimental procedures were approved by the Institutional Animal Care and Use Committee of the Henan Provincial People's Hospital, Zhengzhou University (Experiment Ethical No: HNEECA-2022-20). All methods were carried out in accordance with relevant guidelines and regulations (the Association for Research in Vision and Ophthalmology’s statement) and in compliance with the Animal Research: Reporting of In Vivo Experiments (ARRIVE) guidelines.

### Data collection

The body weight and blood glucose of the two groups of mice were measured after two weeks of adaptation in the circadian rhythm chamber. Tear secretion level was measured as previously described^12^. Briefly, mice were given pilocarpine hydrochloride (a cholinergic agonist for the parasympathetic nerve, 4.5 mg/kg) in saline by intraperitoneal injection. After 10-min recovery, tear volumes were measured using the phenol red thread test (#30059010; Tianjin Jingming New Technology Development Co., Tianjin, China) by carefully placing the thread at the inner canthus of the eye and holding it in place for 20 s. The length (millimeters) of the red portion was measured to evaluate the amount of tear secretion. Animals were anesthetized with 2% isoflurane before euthanasia through cervical dislocation. The ELGs of mice were collected and quickly put into liquid nitrogen for subsequent RNA extraction and RNA sequencing. Total RNA was extracted from ELGs and purified using RNeasy spin column kit (Qiagen, Hilden, Germany). And mRNA was then enriched using Oligo (dT) magnetic beads. The cDNA fragments were amplified by PCR after the first and second cDNA strands being synthesized. The BGISEQ-500 sequencing platform (BGI, Wuhan, China) was used to sequence data libraries. SOAPnuke software (v1.5.2 https://github.com/BGI-flexlab/SOAPnuke) was used to obtain clean reads. Mapping with the reference gene set was conducted using the Bowtie2 software (v2.2.5 http://bowtiebio.sourceforge.net/%20Bowtie2%20/index.shtml) and mapping with the reference genome using the HISAT software (v2.0.4 http://www.ccb.jhu.edu/software/hisat/index.shtml). The raw count data were normalized using the fragments per kilobase per million mapped fragments (FPKM) value.

### Identification of differentially expressed genes between WT and DB mice

Differentially expressed genes (DEGs) were identified by using the DESeq package of R software (version 4.2.0). DEGs were defined as absolute log2 fold change (FC) > 1 and an adjusted *P* value of < 0.05.

### Functional annotation with enrichment analysis

DEGs were subjected to gene ontology (GO) and Kyoto Encyclopedia of Genes and Genomes (KEGG) pathway analysis by using the Database for Annotation, Visualization and Integrated Discovery (DAVID) bioinformatics tool (version 6.8), which to identify the functional roles of the upregulated and downregulated DEGs, respectively. GO enrichment was described from three aspects: biological process (BP), molecular function (MF), and cellular component (CC). Annotated reference gene sets of c5.all.v7.1.symbols.gmt were downloaded from the Molecular Signatures Database.

### Statistical analysis

All statistical analyses were conducted with R software (version 4.2.0) and GraphPad Prism software version 8.0 (GraphPad Software, La Jolla, CA). All mice of each group were randomly separated into a training set (*n* = 14) and testing set (*n* = 6). To further narrow the scope of the candidate marker genes related to T2DM, we adopted the LASSO binary logistic regression model using the training set, which was applied to minimize overfitting and identify the most significant T2DM-associated DEGs in ELGs. The risk-score signature for prediction of T2DM was obtained by multiplying the expression level of each selected marker genes by its corresponding relative regression coefficient weight, as follows:

$$ {\text{risk}}\;{\text{score}} = \mathop \sum \limits_{i = 1}^{N} \beta i \times Ei, $$

where *N* represents the total number of selected marker genes, *βi* represents the coefficient index of each gene calculated by LASSO regression, and *Ei* represents the gene expression value of each gene. The risk score was calculated for each mouse in the training set. Those with a higher risk score than the median score were classified into the high-score group, while those with a lower risk score were classified into the low-score group. Receiver operating characteristic (ROC) curves of the regression model were plotted to evaluate the sensitivity and specificity of the marker genes we screened. The survival package of R software was used to construct a nomogram for the screened marker genes, and the nomogram was assessed by discrimination and calibration. The concordance index (C-index) was calculated to evaluate the efficiency of the nomogram by using the bootstrap method with 1000 resamples. The calibration curve of the nomogram was plotted to observe the relationship between the nomogram prediction probability and the observation rate. The test set was used to verify the discrimination ability of the selected marker genes. All statistical tests were two-sided, and *P* values less than 0.05 were considered statistically significant.

## Results

### General descriptions of the control and T2DM mice

**Figure 1:**
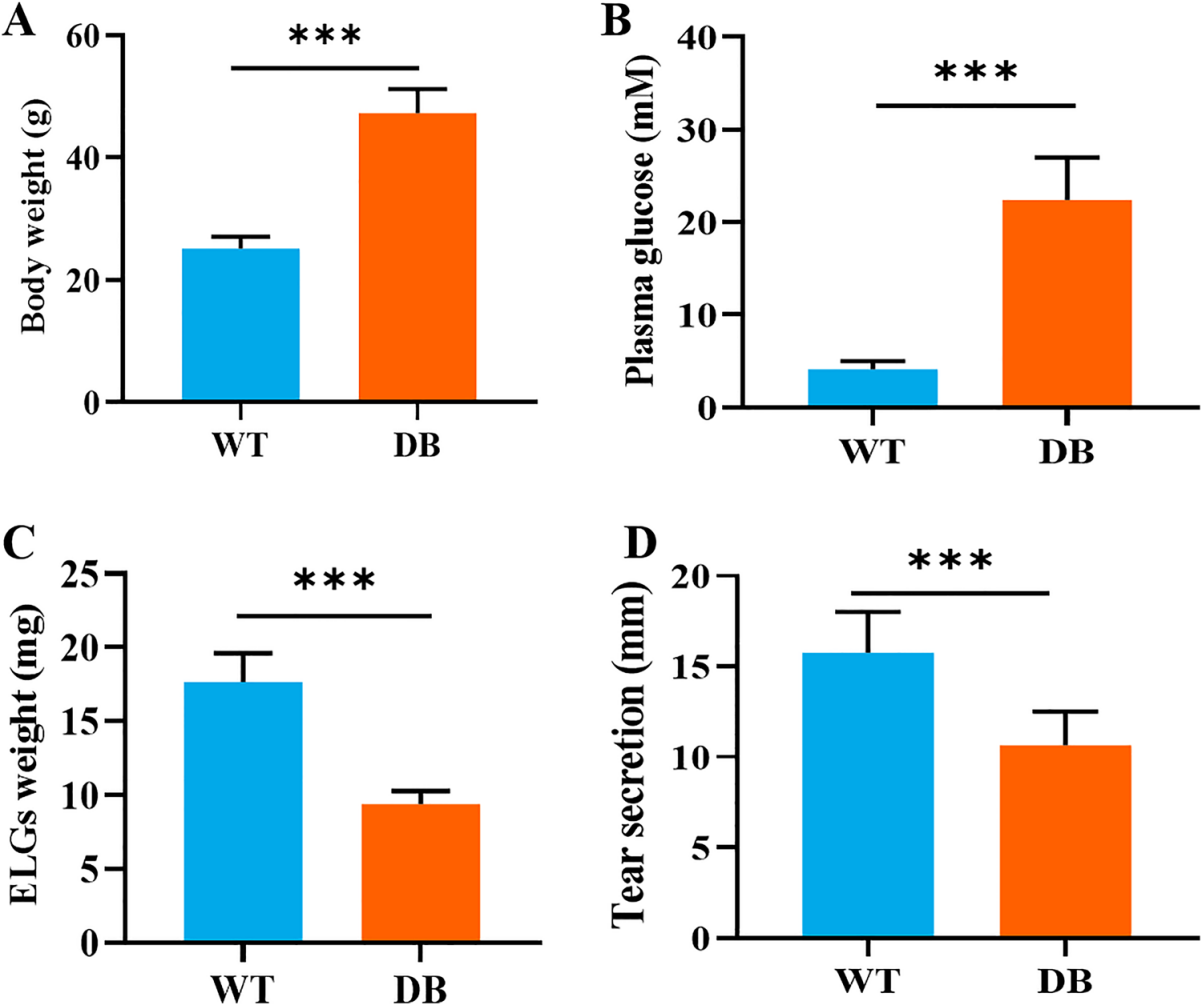
Comparison of general characteristics between WT and DB mice.

The body weight of the WT and DB mice was 25.10 ± 1.94 g and 47.27 ± 3.88 g, respectively. The difference was statistically significant between two groups (*t* = 22.85, *P* < 0.001) (Fig. 1A). There was a statistical difference in the fasting blood glucose level between WT group (4.12 ± 0.85 mmol/L) and DB group (22.40 ± 4.57 mmol/L) (*t* = 17.570, *P* < 0.001) (Fig. 1B). The ELG weight of the WT mice (17.65 ± 1.94 mg) was significantly heavier than that of the DB mice (9.37 ± 0.91 mg) (*t* = 17.280, *P* < 0.001) (Fig. 1C). There were significant statistical differences in level of tear secretion between WT (15.77 ± 2.22 mm) and DB group (10.64 ± 1.87 mm) (*t* = 8.299, *P* < 0.001) (Fig. 1D).

### Screening of DEGs associated with T2DM

**Figure 2:**
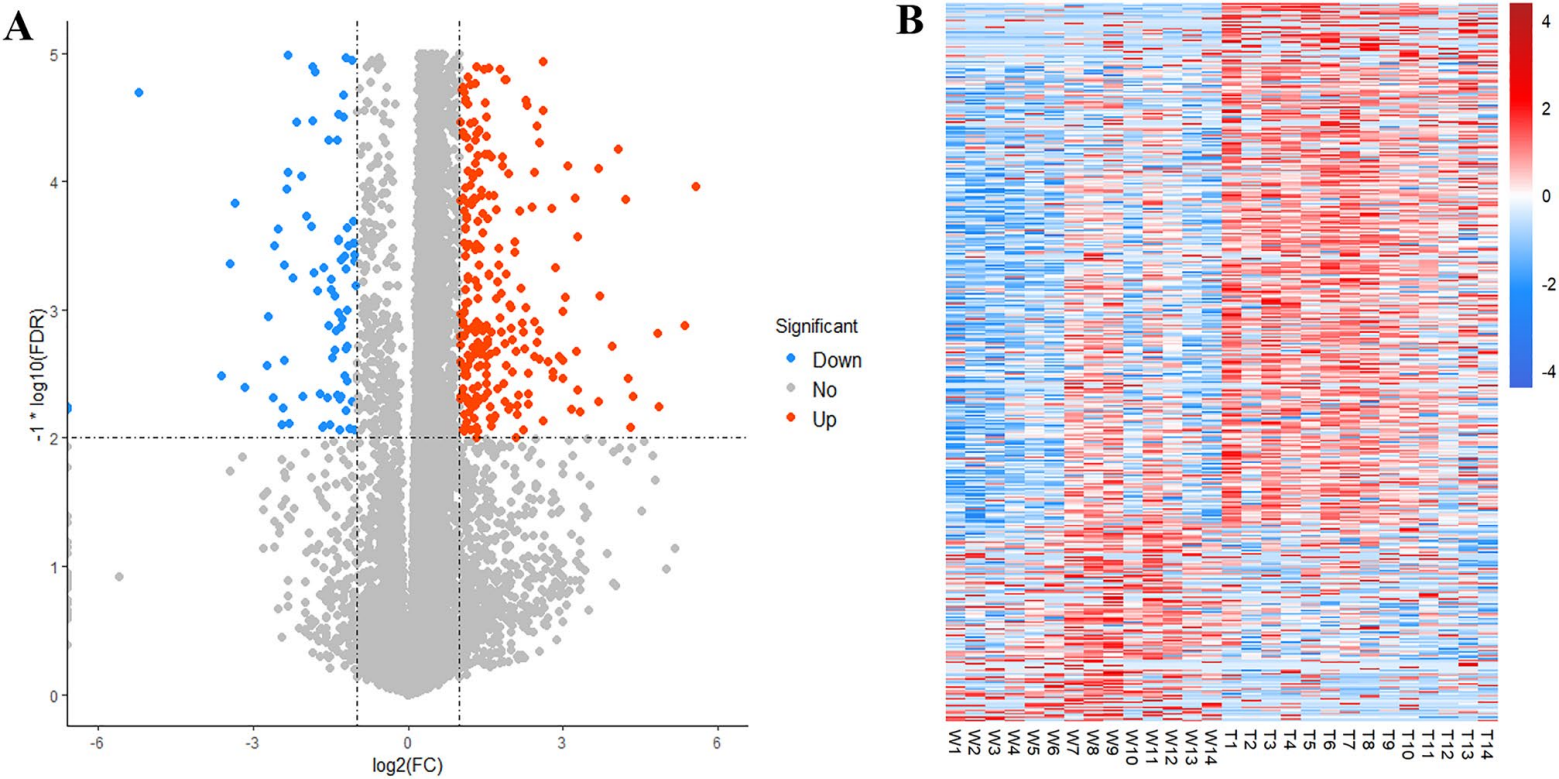
The differentially expressed genes between WT and DB mice. (**A**) The volcano plot of DEGs between two groups. (**B**) The heatmap displays expression levels of 689 DEGs based on the RNA-seq data.

Altogether, 16,677 transcripts were sequenced for WT and DB mice, respectively. Sixteen samples of each group were used as the training set to screen DEGs. Under the conditions of FDR < 0.01 and |log_2_ (FC)|> 1, 689 DEGs were screened. The volcano plot of DEGs between two groups is shown in Fig. 2A, in which, 166 genes were downregulated and 523 genes were upregulated in DB group. Heatmaps display the standardized expression of 689 DEGs (Fig. 2B).

### Biological functions of DEGs associated with T2DM

**Figure 3:**
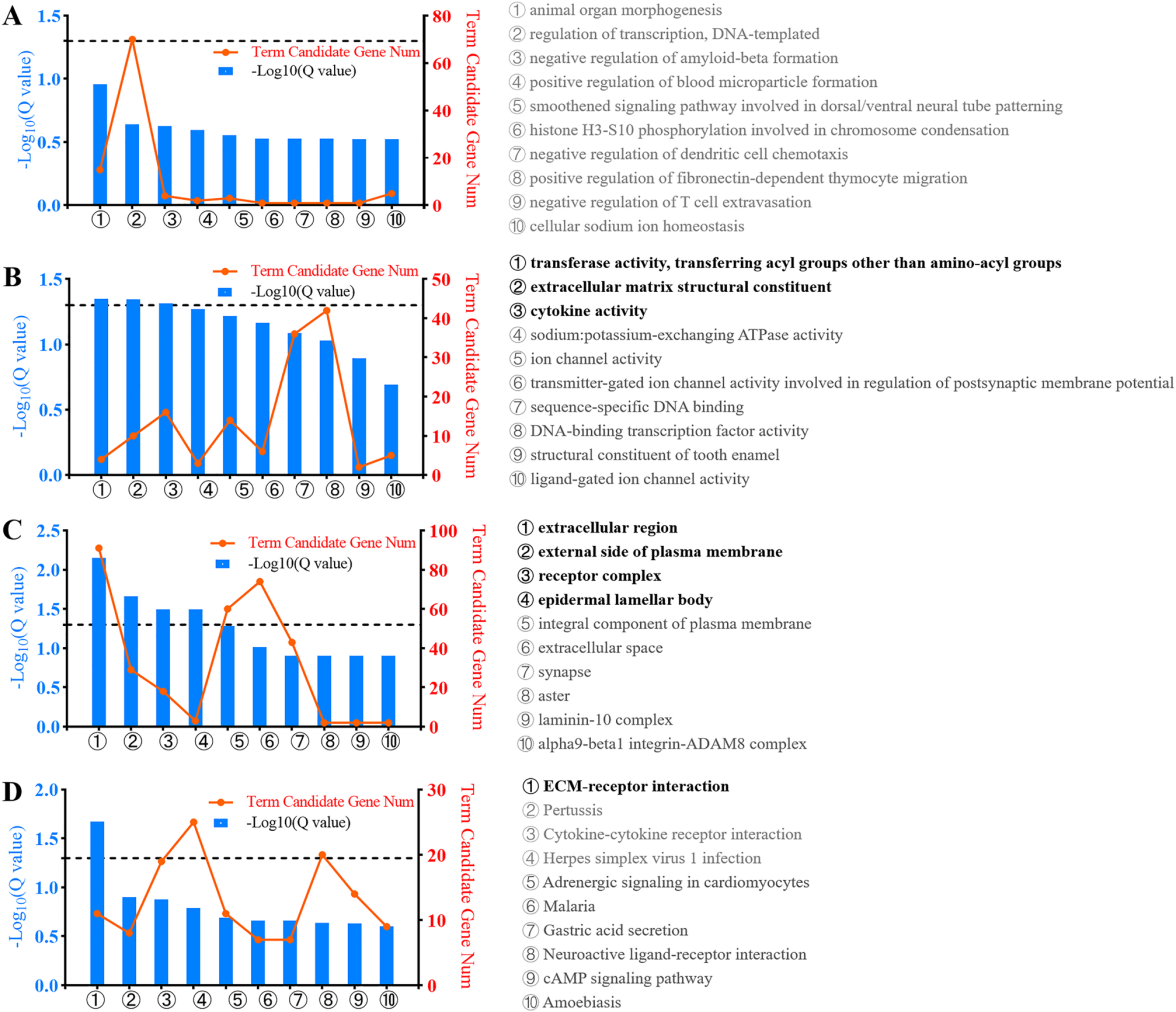
Gene annotation of GO and KEGG pathways enriched in DEGs. (**A**–**C**) The biological processes (**A**), molecular functions (**B**) and cellular components (**C**) of the DEGs; (**D**) Annotation of 689 DEGs from the KEGG pathway database. The text in bold represents the pathway that *Q* < 0.05.

To establish links between DEGs and biological processes, GO annotation and KEGG pathway annotation were performed using the gene2go.gz and KEGG database V81.0 with NCBI RefSeq GCF_000001635.25_GRCm38.p5 as a reference gene set, respectively. The biological processes, molecular functions and cellular components of the DEGs are presented in Fig. 3A–C. No biological process was annotated in these DEGs. Three molecular functions were annotated, including transferase activity, transferring acyl groups other than amino-acyl groups, extracellular matrix structural constituent, and cytokine activity. Four cellular components were annotated, including extracellular region, external side of plasma membrane, receptor complex, and epidermal lamellar body. One KEGG pathway was annotated in these DEGs (Fig. 3D).

### Identification of marker genes related to T2DM using LASSO regression

**Figure 4:**
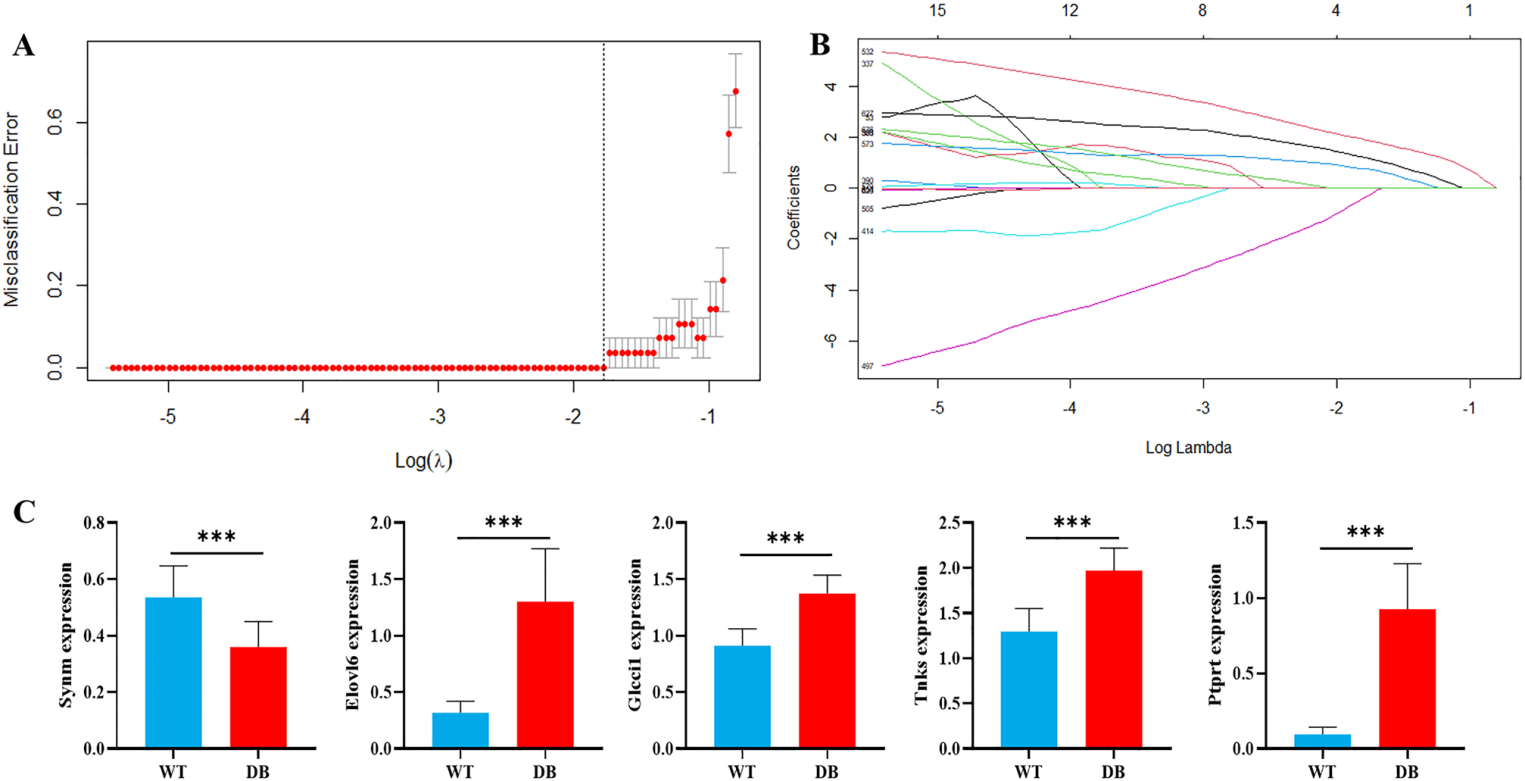
Screening of marker genes related to T2DM based on LASSO regression. (**A**) Cross validation plot for the penalty term. When logλ =  − 0.775, a minimal root mean square error (RMSE) and 5 non-zero coefficients were obtained. (**B**) LASSO regression coefficients over different values of the penalty parameter. (**C**) Expression of the 5 marker genes in WT and DB groups. ***P < 0.001.

Fourteen samples from 20 samples in each group were randomly selected as the training set, and the LASSO regression model was used to screen the marker genes related to T2DM. The 689 DEGs were included in the model for variable screening, the minimum standard was adopted to obtain the value of the parameter λ by tenfold cross-validation. The binomial deviance was computed for the training data as a measure of the predictive performance of the fitted models. Screening of marker genes related to T2DM based on LASSO regression are shown in Fig. 4. The results showed the λ values ranged from 0.00047 to 0.44720. Cross validation plot for the penalty term is shown in Fig. 4A. Plots for LASSO regression coefficients over different values of the penalty parameter are shown in Fig. 4B. The λ value with a minimal root mean square error (RMSE) was confirmed as 0.1683709 where the optimal lambda resulted in 5 non-zero coefficients. The marker genes identified by LASSO regression included *Synm*, *Elovl6*, *Glcci1*, *Tnks* and *Ptprt*. The expression of the 5 marker genes in WT and DB group is shown in Fig. 4C. The 5 marker genes were divided into low expression (Low_exp_) and high expression (High_exp_) groups according to the median of the expression. Logistic regression was conducted to estimate the impact of each marker gene on ELGs of T2DM mice. Table 1 displays the results of the logistic regression analysis of 5 marker genes. Lower expression of *Synm* and higher expression of *Elovl6*, *Glcci1*, *Tnks*, and *Ptprt* may increase the risk of T2DM.

**Table 1:** Results of the logistic regression analysis of 5 marker genes.

	WT		DB		*χ*^2^	*P*	*OR* (95% *CI*)
*Synm*							
Low_exp_	2	14.3%	11	78.6%	11.631	0.001	0.045(0.006–0.325)
High_exp_	12	85.7%	3	21.4%			
*Elovl6*							
Low_exp_	13	92.9%	1	7.1%	20.571	< 0.001	169.000(9.521–2999.916)
High_exp_	1	7.1%	13	92.9%			
*Glcci1*							
Low_exp_	12	85.7%	0	0.0%	21.000	< 0.001	0.143(0.040–0.515)
High_exp_	2	14.3%	14	100.0%			
*Tnks*							
Low_exp_	12	85.7%	0	0.0%	21.000	< 0.001	0.143(0.040–0.515)
High_exp_	2	14.3%	14	100.0%			
*Ptprt*							
Low_exp_	14	100.0%	0	0.0%	28.000	< 0.001	–
High_exp_	0	0.0%	14	100.0%			

### Efficiency of marker genes screened by LASSO model

**Figure 5:**
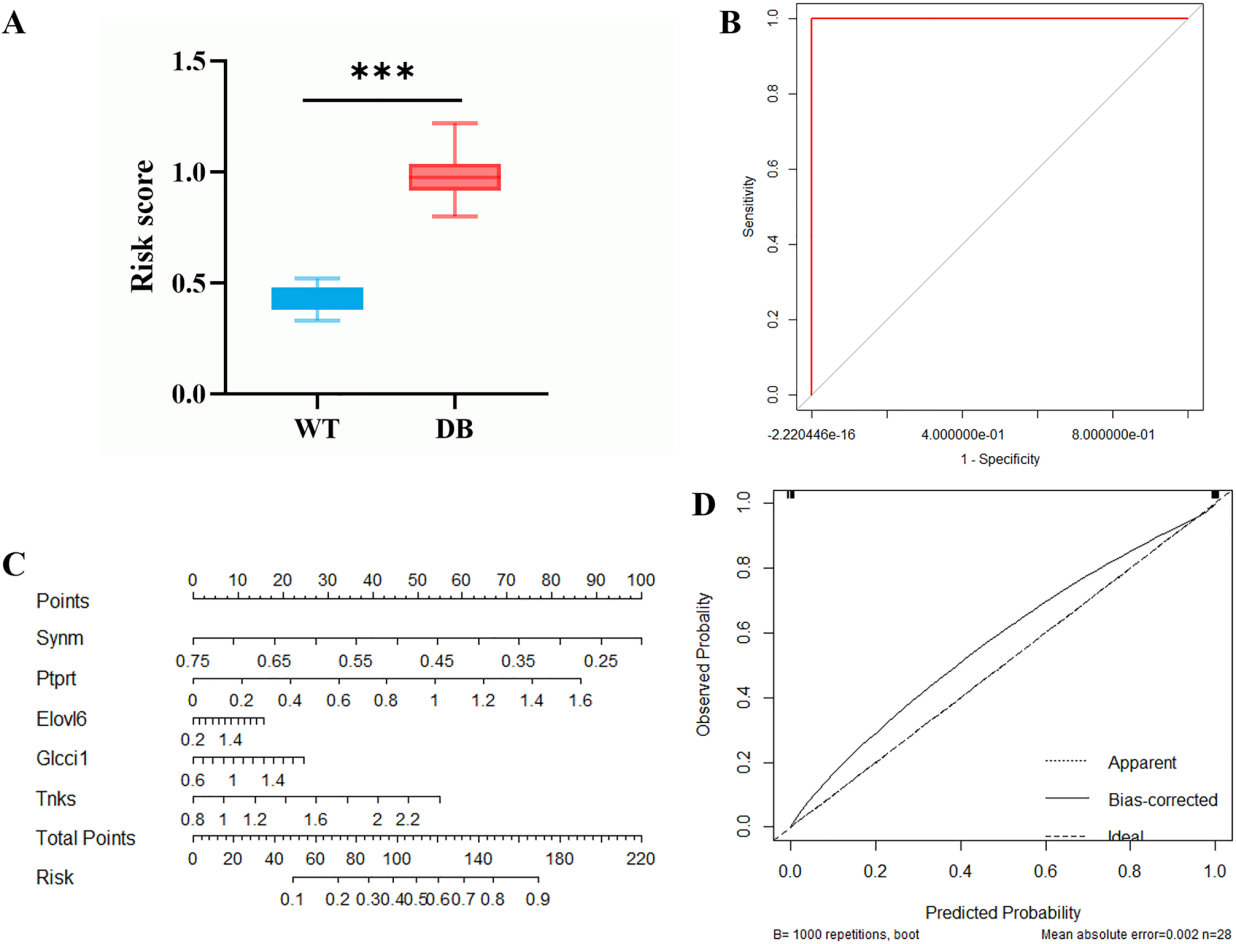
Efficiency of marker genes screened by LASSO model using the training set. (**A**) The high TG-score group had a higher risk of T2DM than the low TG-score group. (**B**) The ROC curve of the marker genes-model. The AUC of the model was 1.000(1.000–1.000). (**C**) The nomogram of LASSO regression. (**D**) The calibration curve of the marker genes-model. The C-index and the robust C-index were 1.000 and 0.999, respectively. ***P < 0.001.

We used the minimal λ to build a T2DM-related gene score which we named the “TG-score”. The TG-score =  − 0.1884 * Synm_exp_ + 0.0022 * Elovl6_exp_ + 0.1800 * Glcci1_exp_ + 0.2555 * Tnks_exp_ + 0.3499 * Ptprt_exp_. Next, we attempted to further determine the value of TG-score by predicting the T2DM patients. We divided patients into high and low TG-score groups with the best cut-off value of 0.60. We found that the high TG-score group had a higher risk of T2DM than the low TG-score group (Fig. 5A).

The regression model was constructed with 5 marker genes as independent variables to obtain the probability that each sample was identified as T2DM. Taking the probability of each sample as the test variable and grouping WT and DB mice as the state variable, ROC analysis was carried out and a ROC curve was obtained. The area under receiver operating curve (AUC) of the model was 1.000(1.000–1.000), which indicated that the efficiency of the model was good. The ROC curve of the marker genes-model is shown in Fig. 5B.

A nomogram was created using LASSO regression analysis, which to estimate the probability of T2DM (Fig. 5C). The calibration curve was used to describe the prediction value of the nomogram, and the 45° line indicates the actual diagnostic grouping. The C-index and the robust C-index were 1.000 and 0.999, respectively. The results for identifying T2DM mice showed that the nomogram-predicted T2DM closely matched the best prediction performance (Fig. 5D), indicating that these 5 marker genes screened by LASSO regression have a significant predictive value for identifying T2DM. Collectively, our results indicated a good performance of the 5-marker genes for T2DM.

### Validation of the marker genes screened by LASSO model

**Figure 6:**
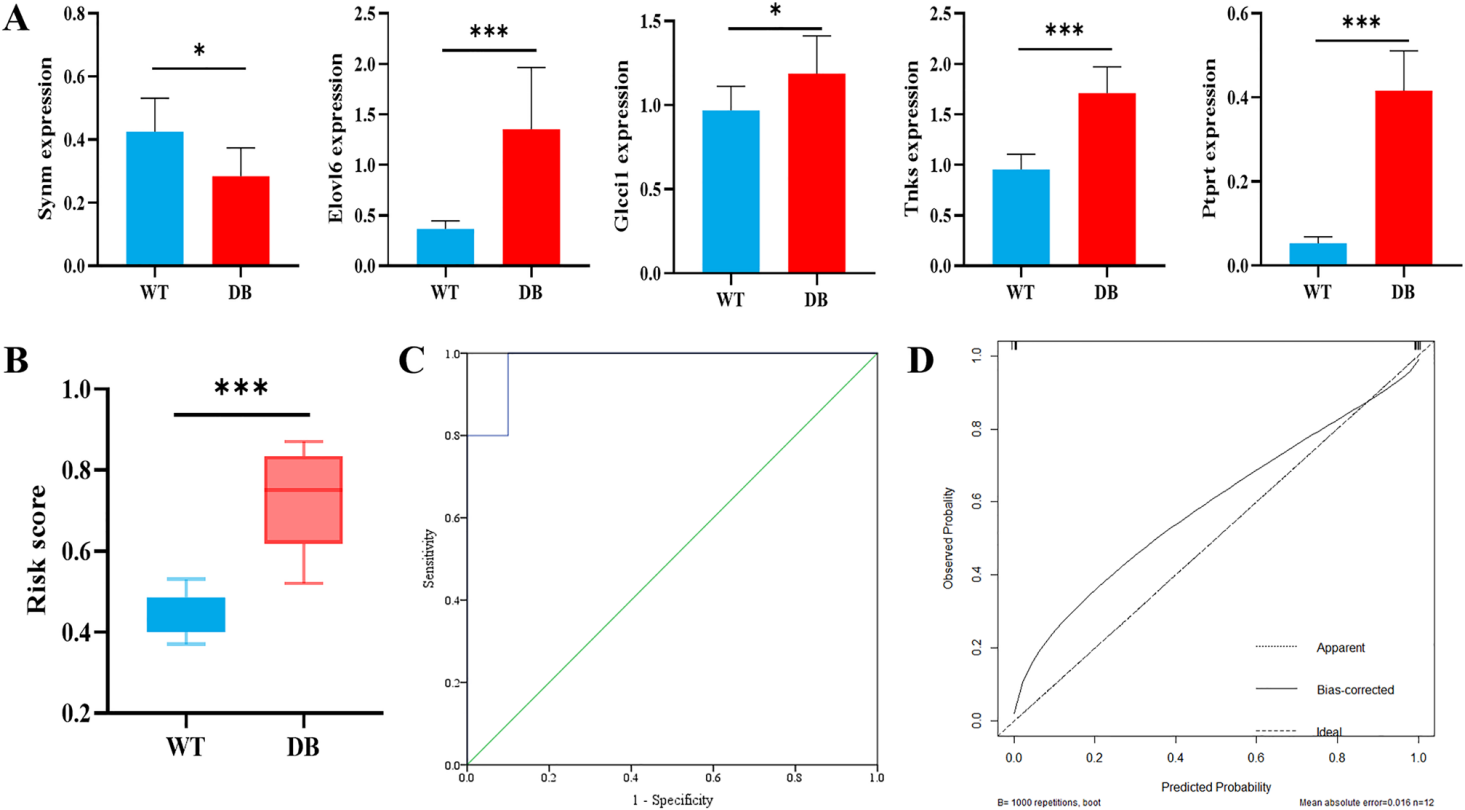
Validation of the marker genes screened by LASSO model using the test set. (**A**) Expression of the 5 marker genes in the test set. (**B**) Similar to the findings in the training set, the test set showed the high TG-score group had a higher risk of T2DM than the low TG-score group. (**C**) The ROC curve of the marker genes-model in the test set. The AUC of the model was 0.980(0.929–1.000). (**D**) The calibration curves for the possibility of T2DM in the test set. The C-index and the robust C-index were 1.000 and 0.978, respectively. ***P < 0.001.

The marker genes were further validated by the test set (n = 6), which to evaluate the robustness of the LASSO model. The expression of the 5 marker genes in the test set is shown in Fig. 6A. Similar to the findings in the training set, the test set was consistently stratified into the high TG-score group with a higher risk of T2DM and the low TG-score group with a lower risk of T2DM (*P* < 0.001) (Fig. 6B). The probability of identifying T2DM in the test samples was obtained using the 5 marker genes. Similar to the performance in the training set, the AUC of the probability value was 0.980(0.920–1.000) in our validation dataset (Fig. 6C). The nomogram calibration curves for the possibility of T2DM displayed obvious concordance between the predicted results and observations in the test set (Fig. 6D). The C-index and the robust C-index were 1.000 and 0.978, respectively. The results show that the marker genes screened by LASSO model performed best in both the training and the validation sets.

### Relationship between T2DM marker genes and lacrimal gland function

**Figure 7:**
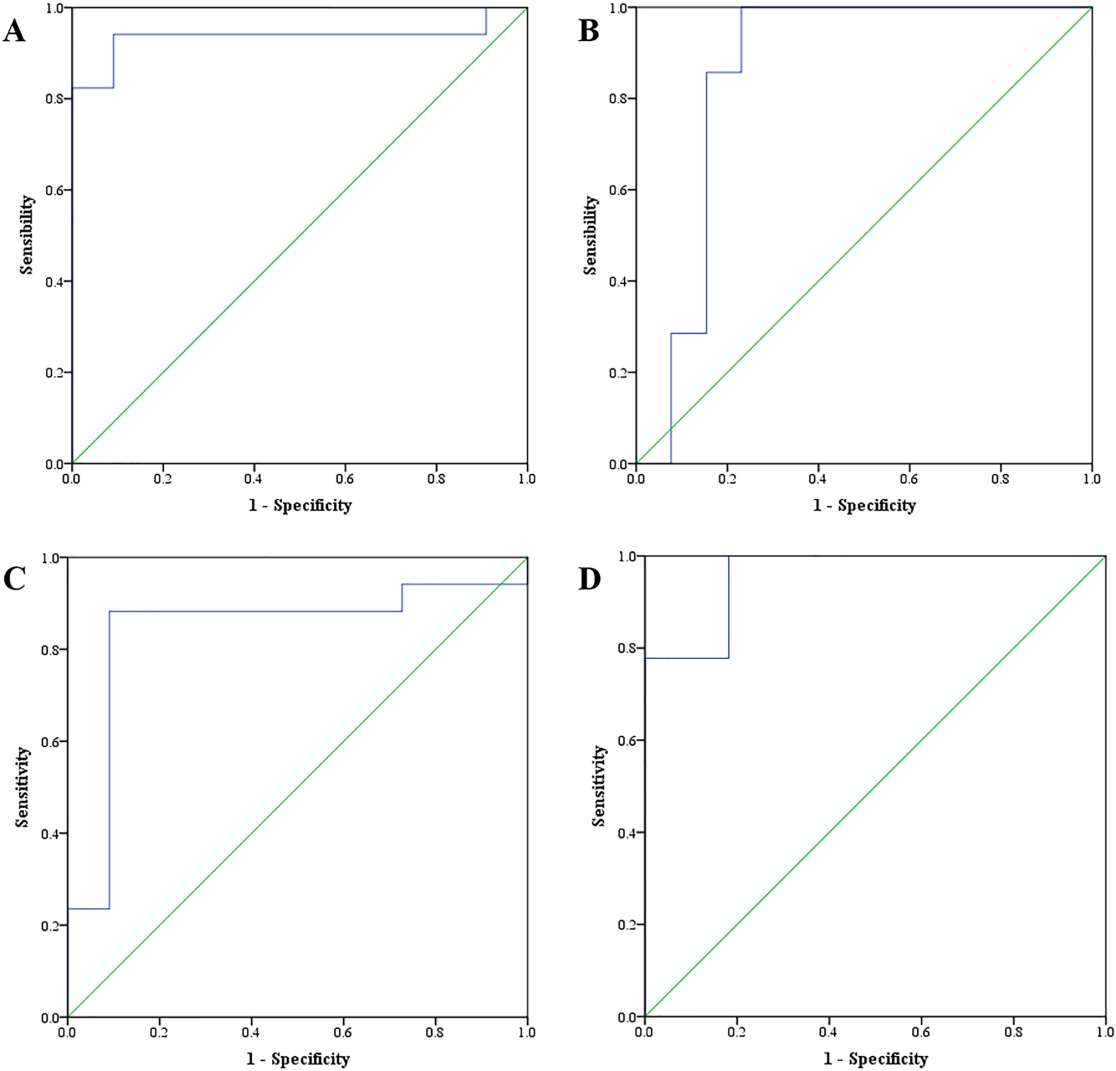
ROC curves of the LASSO model in predicting ELG function. (**A**) The ROC curve of the LASSO model in predicting ELG weight in the training set; (**B**) The ROC curve of the LASSO model in predicting ELG weight in the test set; (**C**) The ROC curve of the LASSO model in predicting the level of tear secretion in the training set; (**D**) The ROC curve of the LASSO model in predicting the level of tear secretion in the test set.

In order to explore the relationship between expression of marker genes and lacrimal gland function, the correlation coefficient between weight of lacrimal gland, as well as tear secretion and expression of 5 marker genes was calculated by Spearman correlation analysis (Table 2). The results indicated expression of *Synm* was positively correlated with the weight of lacrimal gland. While, the expression of *Elovl6*, *Glcci1*, *Tnks* and *Ptprt* was negatively correlated with the weight of lacrimal gland and tear secretion. The efficacy of the TG-score of the LASSO model in predicting ELG function (ELG weight and the level of tear secretion) was analyzed with adjusting the expression of leptin receptor gene (*Lepr*). The ROC curves of the LASSO model in predicting ELG weight and the level of tear secretion are shown in Fig. 7. Results show that the AUC of the LASSO model in predicting ELG weight was 0.936(0.831–1.000) in the training set, and 0.857(0.681–1.000) in the test set (Fig. 7A,B). The AUC of the LASSO model in predicting the level of tear secretion was 0.840(0.668–1.000) in the training set, and 0.960(0.882–1.000) in the test set (Fig. 7C,D). These findings suggest abnormal expression of marker genes is related to lacrimal gland atrophy and dry eye in mice.

**Table 2:** Relationship between expression of marker genes and lacrimal gland function.

	*Synm*	*Elovl6*	*Glcci1*	*Tnks*	*Ptprt*
Weight of lacrimal gland					
*r*	0.386	− 0.849	− 0.761	− 0.767	− 0.882
*P*	0.007	< 0.001	< 0.001	< 0.001	< 0.001
Tear secretion					
*r*	0.221	− 0.791	− 0.562	− 0.727	− 0.862
*P*	0.131	< 0.001	< 0.001	< 0.001	< 0.001

## Discussion

Owing to the rising prevalence of T2DM, its high economic cost and serious complications, primary prevention and timely intervention are essential in preventing or delaying the onset of T2DM. Genome wide association studies have identified more than 400 genetic variations associated with T2DM, which together account for about 15–18% of the occurrence of T2DM. Marker genes are valuable for timely identification and the implementation of effective intervention in a high-risk population. In this study, we screened five marker genes that were highly related to T2DM in the lacrimal gland of db/db mice using LASSO regression, including *Synm*, *Elovl6*, *Glcci1*, *Tnks* and *Ptprt*. The results of multiple validation showed the model had good efficiency and stability for identifying T2DM, indicating that these five selected genes were highly correlated with T2DM and could be used as marker genes. In addition, we found that the expression of these genes was also significantly related to the lacrimal gland function, and the incidence of dry eyes in mice with abnormal expression was higher.

In recent years, the prediction and screening models of T2DM have become a research hotspot. Most of the models are based on age, gender, disease history, behavior habits and other indicators. In addition, DM is also closely related to hereditary factors. However, previous prediction models seldom consider the role of genetic factors in disease occurrence. In addition, there are few reports about the genes related to T2DM in lacrimal gland tissue. LASSO regression is characterized by variable screening and complexity adjustment while fitting the generalized linear model. It has unique advantages in dealing with high-dimensional data, even data whose dimension is much larger than the sample size. Adjusting the complexity of the model by control λ, can not only make the mean square error of the prediction model reach an ideal level, but can also avoid the overfitting of the model, which is especially suitable for the screening of marker genes in transcriptome data. In this study, five marker genes related to T2DM were selected by LASSO regression. The results shows that there is a certain statistical correlation between T2DM and marker genes, and provided a new idea for prediction and gene therapy of high-risk groups in the future.

The results showed that the expression of *Synm* in the lacrimal gland of T2DM mice was lower than that of control mice. The study of Juvinao-Quintero et al.^13^ indicated cg16765088 (near *SYNM*) of methylated CpG sites was associated with T2DM in European populations. *Synm* is located on chromosome 15, and the protein encoded by this gene is a member of the intermediate filament family. Synemin (*SYNM*) is an intermediate filament protein copolymerized with desmin and vimentin, which is expressed in gastrointestinal tract, bladder, brain, ovary and so on. *SYNM* deficiency will affect the copolymerization with vimentin^14^, and vimentin deficiency has been reported to affect vascular response^15^. Studies have shown that *SYNM* decreases after arterial injury^16^. The effect of decreased expression of *Synm* on vascular reactivity may be one of the causes of T2DM and its vascular complications.

The long chain fatty acid family member 6 (*Elovl6*) gene located on chromosome 4q25 encodes a microsomal enzyme that catalyzes the extension of saturated and monounsaturated fatty acids containing 12, 14 and 16 carbons^17,18^. Studies have shown that overexpression of *Elovl6* can lead to dysfunction of the β cell^19^. *Elovl6* deficiency increases insulin sensitivity and reduces insulin resistance^20^. The knockout of *Elovl6* limits the extension of palmitate to stearate, thus desaturating palmitate to palmitoleic acid, which is a potential low-fat toxic fatty acid. This leads to palmitic acid-induced endoplasmic reticulum stress and attenuation of pancreas β apoptosis^19^. Therefore, the reduced expression of *Elovl6* may be beneficial to protective role of β cell^21^. The results of this study showed that the expression of *Elovl6* in the lacrimal gland of T2DM mice was higher than that of control mice. Matsuzaka's research demonstrates that manipulating fatty acid components by blocking *Elovl6* can prevent insulin resistance, impaired insulin secretion and obesity-related diseases^22^. These facts suggest that *Elovl6* may be involved in the development of T2DM, and limiting the expression or activity of *Elovl6* may be a new method for the treatment of T2DM.

*Glcci1* gene is expressed in many tissues and is considered to be an early marker of glucocorticoid induced apoptosis^23^. In cultured thymocyte lines, upregulation of glucocorticoid receptor dependent *GLCCI1* is associated with glucocorticoid induced proapoptotic events^24^. The change of *Glcci1* expression may affect the role of glucocorticoids in regulating glucose and fat metabolism through this mechanism. Kim et al. found that the expression of *Glcci1* in the glomerulus of hyperglycemic rats decreased significantly, but increased in diabetic rats treated with wortmannin^25^. The present study showed that compared with the control group, the expression of *Glcci1* in the lacrimal gland of T2DM group was upregulated. This may be due to the tissue-specific expression of *Glcci1*. The mechanism of *Glcci1* gene affecting T2DM in different organs and tissues needs further study.

Tankyrases (TNKSs) are members of the poly ADP-ribose polymerase (PARP) family. They catalyze post-translational modification of proteins by transferring the ADP ribose portion of NAD+ to the target protein^26^. The activity of TNKSs affects the function and stability of target proteins, thereby regulating a variety of cellular processes, including cell differentiation and energy metabolism. In humans, *Tnks* is located on chromosome 8p23.1, which is the susceptibility site of T2DM. The mutation of *Tnks* is related to early-onset obesity. This study showed that the expression of *Tnks* in the lacrimal gland of T2DM mice was higher than that of control mice. Other studies have shown that the specific destruction of *Tnks1* activity in adipocytes can enhance glucose tolerance and insulin sensitivity in female mice. Inhibition of TNKS1 (a subtype of TNKSs) with TNKS specific inhibitors can reduce fasting blood glucose and improve insulin sensitivity^27^. Wang et al. found that TNKS inhibition reduced the body weight, abdominal fat content and serum cholesterol level of db/db mice, by driving the transcription reprogramming of PGC-1α in muscle and white adipose tissue^26^. These results suggest that specific TNKS activity inhibitor may become a potential drug for the treatment of obesity and T2DM.

The protein encoded by *Ptprt* gene is a member of protein tyrosine phosphatase (PTP) family. PTPs are signaling molecules that regulate a variety of cellular processes, including cell growth, differentiation and carcinogenic transformation. *PTPRT* can inhibit lung cancer, breast cancer and other malignant tumors by regulating JAK-STAT pathway and inhibiting transcription activator-3 (STAT3)^28,29^. *Ptprt* knockout mice have upregulated STAT3 phosphorylation in the hypothalamus, are resistant to obesity induced by a high-fat diet, and show improved peripheral insulin sensitivity and reduced blood glucose^30^. The results of this study showed that the expression of *Ptprt* was upregulated in the lacrimal gland of T2DM mice. These results suggest that *Ptprt* may have an effect on obesity and diabetes by regulating STAT3 signal.

In this study, five marker genes highly related to T2DM were screened by LASSO regression, which provided a scientific basis for the prediction and gene therapy of T2DM. However, this research has some limitations. First, in order to exclude the impact of sex hormones, only male mice were used in this study. Whether these five marker genes can also effectively identify T2DM in the lacrimal gland of female mice remains to be further studied. Second, because gene expression is tissue-specific, this study only collected lacrimal gland samples for RNA sequencing. The five marker genes may not be applicable to other tissues and organs. In addition, sampling of lacrimal gland and gene quantification are not easy to be applied in practice. In the future, we will continue to explore the marker genes in tissues that are easy to acquire, such as blood or hair. Third, this was a cross-sectional study, rather than a prospective study, the results of which only indicated a statistical association between five marker genes and T2DM. Thus, it is not possible to infer whether there is a causal relationship between these marker genes and T2DM. In addition to T2DM and marker genes, the differences in lacrimal gland function between the two groups in this study may also be related to a deficient leptin signal in db/db mice and various other factors. Finally, although the internal validation showed the results were robust, external validation was not carried out. In the future, multi-center external validation studies will be conducted to further study the mechanism of marker genes affecting the occurrence and development of T2DM.

## Conclusion

In the lacrimal gland of db/db mice, *Synm*, *Elovl6*, *Glcci1*, *Tnks* and *Ptprt* can be used as marker genes of T2DM. Mice with abnormal expression have a higher prevalence of T2DM and dry eye. These findings provide an insight for the study of dry eyes caused by T2DM, and will be explored in other organ tissues such as blood and hair in the future. The discovery of the marker genes provides a new direction for the gene therapy of T2DM and complication caused by T2DM in clinical practice, by transferring exogenous normal genes into the target cells to correct or compensate for abnormal marker genes.

## Data Availability

All relevant data are included in the papers. Please contact the corresponding author for additional information regarding data access.

## Abbreviations

AUC: Area under receiver operating curve
BP: Biological process
CC: Cellular component
C-index: Concordance index
DAVID: Database for annotation, visualization and integrated discovery
DM: Diabetes mellitus
DEGs: Differentially expressed genes
ELG: Extraorbital lacrimal gland
FC: Fold change
FPKM: Fragments per kilobase per million mapped fragments
GO: Gene ontology
High_exp_: High expression
KEGG: Kyoto encyclopedia of genes and genomes
LASSO: Least absolute shrinkage and selection operator
Low_exp_: Low expression
MF: Molecular function
PARP: Poly ADP-ribose polymerase
PTP: Protein tyrosine phosphatase
ROC: Receiver operating characteristic
RMSE: Root mean square error
TNKSs: Tankyrases
T2DM: Type 2 diabetes mellitus
WT: Wild-type mice
